# Water Extract of Ashwagandha Leaves Has Anticancer Activity: Identification of an Active Component and Its Mechanism of Action

**DOI:** 10.1371/journal.pone.0077189

**Published:** 2013-10-10

**Authors:** Renu Wadhwa, Rumani Singh, Ran Gao, Navjot Shah, Nashi Widodo, Tomoko Nakamoto, Yoshiyuki Ishida, Keiji Terao, Sunil C. Kaul

**Affiliations:** 1 National Institute of Advanced Industrial Science & Technology (AIST), Tsukuba, Ibaraki, Japan; 2 Department of Biology, Faculty of Mathematics and Natural Sciences, Brawijaya University, Malang, Indonesia; 3 CycloChem Co., Ltd. KIBC No. 654 5-5-2 Minamicho Minatoshima Chuo-ku, Kobe City, Japan; Cairo University, Egypt

## Abstract

**Background:**

Cancer is a leading cause of death accounting for 15-20% of global mortality. Although advancements in diagnostic and therapeutic technologies have improved cancer survival statistics, 75% of the world population live in underdeveloped regions and have poor access to the advanced medical remedies. Natural therapies hence become an alternative choice of treatment. Ashwagandha, a tropical herb used in Indian Ayurvedic medicine, has a long history of its health promoting and therapeutic effects. In the present study, we have investigated an anticancer activity in the water extract of Ashwagandha leaves (ASH-WEX).

**Methodology/Principal Findings:**

Anticancer activity in the water extract of Ashwagandha leaves (ASH-WEX) was detected by *in* *vitro* and *in* *vivo* assays. Bioactivity-based size fractionation and NMR analysis were performed to identify the active anticancer component(s). Mechanism of anticancer activity in the extract and its purified component was investigated by biochemical assays. We report that the ASH-WEX is cytotoxic to cancer cells selectively, and causes tumor suppression *in* *vivo*. Its active anticancer component was identified as triethylene glycol (TEG). Molecular analysis revealed activation of tumor suppressor proteins p53 and pRB by ASH-WEX and TEG in cancer cells. In contrast to the hypophosphorylation of pRB, decrease in cyclin B1 and increase in cyclin D1 in ASH-WEX and TEG-treated cancer cells (undergoing growth arrest), normal cells showed increase in pRB phosphorylation and cyclin B1, and decrease in cyclin D1 (signifying their cell cycle progression). We also found that the MMP-3 and MMP-9 that regulate metastasis were down regulated in ASH-WEX and TEG-treated cancer cells; normal cells remained unaffected.

**Conclusion:**

We provide the first molecular evidence that the ASH-WEX and TEG have selective cancer cell growth arrest activity and hence may offer natural and economic resources for anticancer medicine.

## Introduction

Cancer defines a large group of diseases originating from uncontrolled cell division in any part of the body. About 5% of all cancers are strongly hereditary and the others, arising from the internal and external environmental impact, show increase in incidence with increasing age. Cancer treatment involves surgery, radiation, chemotherapy and targeted therapy, that are often complicated by their secondary effect(s) in the immune response. Metastasis, a cancer that has spread from the part of the body where it started (the primary site) to other parts of the body, is most difficult to treat by these conventional ways. In order to prioritize cancer research to develop new ways to treat cancer and improve patient’s quality of life, extremely expensive drug screenings and drug designing have been conducted worldwide. However, the therapeutic power of natural herbs remains underexplored, in spite of the fact that many herbs are affordable and have a long history of human use. 

Ashwagandha (*Withania somnifera*), also known as Winter cherry or Indian ginseng, is a proud herb of Ayurveda, Indian traditional home medicine. Many toxicological studies have demonstrated that Ashwagandha, in its reasonable dose, is a non-toxic, safe and edible herb. It is often categorized amongst the world’s most renowned herbs, such as Korean ginseng, Chinese milkvetch root, female ginseng, reishi mushroom, South American suma and Japanese green tea, and is commonly used in herbal formulations prescribed for general tonic to increase energy and stamina, improve overall health and longevity and to cure conditions such as, inflammation, stress, cardiovascular dysfunction, immune-response and cancer [1-26]. In spite of a long history of its use for health-promoting and therapeutic effects, there are very few laboratory studies on the mechanism of its actions.

Extracts, derived from Ashwagandha root or the whole plant, have been shown to possess anticancer activity that operates through diverse yet converging pathways. Administration of Ashwagandha extract was found to reduce skin carcinogenesis induced by DMBA (dimethyl benzanthracene) through enhancement of anti-oxidant enzymes such as glutathione peroxide and catalase [1]. In another study, Ashwagandha root extract inhibited carcinogen-induced gastric and skin tumorigenesis in Swiss albino mouse model [2]. Several studies have reported the induction of apoptosis, cell cycle arrest, inhibition of angiogenesis and metastasis in response to the treatment with withaferin A, a major phytochemical present in the roots and leaves of Ashwagandha [3-8]. Other pharmacological effects of Ashwagandha include modulation of immune function [9-11], cardioprotection from ischemia and reperfusion injury [12], preventive and therapeutic potential for neurodegenerative diseases [13-20], antibacterial [21-23] and anti-inflammatory activities [9,24-26]. Ashwagandha derived withanolides have been shown to suppress lipopolysaccharide-induced production of inflammatory cytokines and nuclear factor, NF-kB [27-29]. Downregulation of proinflammatory cytokines and a reciprocal upregulation of p38MAPK, PI3K, caspase 6 and cyclin D1 were observed in prostate cancer cells treated with Ashwagandha extract [30]. 

Ashwagandha is rich in phytochemicals including withanamides, withanolides, withanosides, withanolide glycosides, steroidal saponins and lignanamides [31-33]. The major constituents of alcoholic extracts of Ashwagandha are steroidal alkaloids and lactones, known as withanolides [34]. Amongst these, withaferin A and withanone have been demonstrated to be cytotoxic to human cancer cells [35-40]. We have earlier demonstrated that the alcoholic extract (i-Extract) of Ashwagandha leaves and its component withanone cause selective growth arrest of cancer cells through the activation of p53 and ROS signaling pathways [38,40-42]. 

The present study was designed to investigate the effect of water extract of Ashwagandha leaves (ASH-WEX) on normal and cancer cell proliferation. Preparation of such extract is both eco friendly as well as bio friendly because the plant is not sacrificed and organic solvents are replaced with easy, economic, convenient and safe alternative i.e., water.

## Results and Discussion

**Figure 1:**
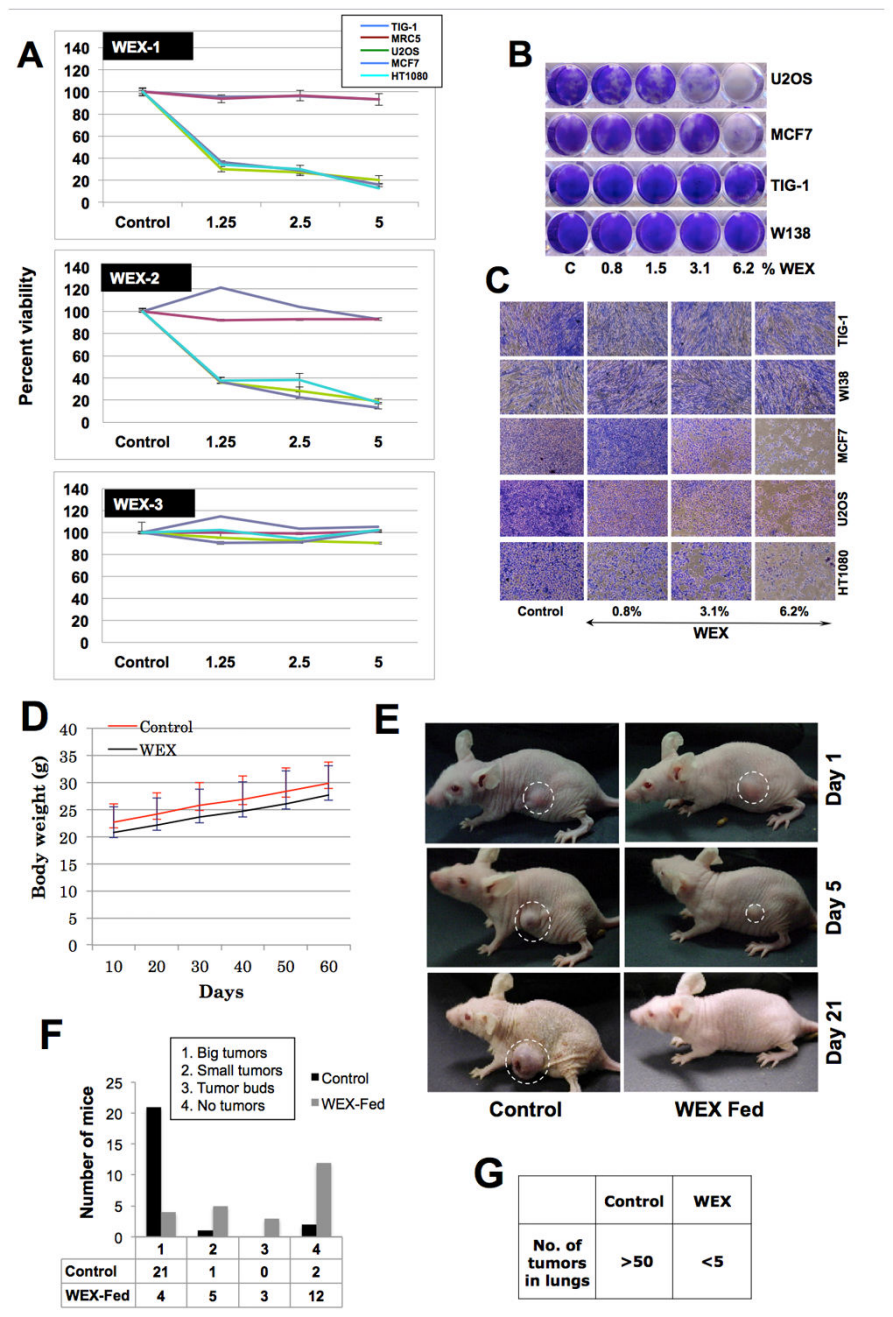
Identification of cancer cell specific cytotoxicity in the water extract of Ashwagandha leaves by *in* *vitro* and *in* *vivo* assays. (**A**) Human normal and cancer cells were treated with three kinds (prepared in different ways as described in materials and methods) of water extracts, WEX-1 (ASH-WEX-1) WEX-2 (ASH-WEX-2) and WEX-3 (ASH-WEX-3). WEX-1 and WEX-2 were predominantly cytotoxic to cancer cells. WEX-3 did not show any cytotoxicity. (**B** and **C**) Human cancer and normal cells treated with serial doses of ASH-WEX and stained with crystal violet showed toxicity to cancer cells. (**D**) Mice fed with WEX (500 mg/Kg body weight) every alternate day did not show any loss in body weight compared to control mice. (**E** and **F**) Nude mice with HT1080 subcutaneous xenograft when fed with WEX showed significant inhibition of tumor progression as well as lung metastasis (**G**). ASH-WEX has been labeled as WEX in all the figures.

 Human normal (TIG-1, WI-38 and MRC5) and tumor derived (U2OS, MCF7 and HT1080) cells were treated with indicated ASH-WEX-1, ASH-WEX-2 and ASH-WEX-3 (ASH-WEX prepared by three different protocols) for 48 h. As shown in Figure 1A, ASH-WEX-1 and ASH-WEX-2 were cytotoxic to cancer cells; normal cells remained unaffected. In contrast to ASH-WEX-1 and ASH-WEX-2, ASH-WEX-3, prepared by autoclaving the leaves-water slurry, did not kill cancer cells suggesting that the cytotoxic component is inactivated by high pressure heating treatment. In order to confirm these results, we performed comparative assays in 48- and 24-well culture dishes. Cells treated with ASH-WEX-1 (henceforth called ASH-WEX) were stained with Crystal Violet for 48 h post-treatment. As shown in Figures 1B and 1C, the treatment of normal and cancer cells with serial doses of ASH-WEX revealed its cytotoxicity to human cancer cells at doses 0.8% and above; normal human cells (MRC5, TIG-1 and WI-38) remained unaffected (Figure 1A-C). These findings suggested that the ASH-WSX has selective cancer cell killing activity, very similar to the alcoholic extract of Ashwagandha leaves [38]. Since the anticancer activity in water extract could be extremely beneficial for human consumption, we extended the study to examine the (i) toxicity assays in mice and (ii) *in vivo* anti-tumor test in nude mice subcutaneous xenograft and tail vein metastasis models. 

 As shown in Figure 1D, mice fed with 500 mg of ASH-WEX/Kg body weight on every alternate day for 60 days did not show toxicity in terms of change in body weight and physical activity. After implanting HT1080 subcutaneous xenograft and tail vein injections, the nude mice were fed with ASH-WEX 250 mg/Kg body weight every alternate day and the tumor formation was monitored. In about 20 days, out of 24 control mice, 21 showed big tumor (Figures 1E and 1F), one showed small tumor and two showed no tumors. ASH-WEX fed mice, on the other hand, showed strong tumor suppression. In this group, out of 24 mice, 12 showed no tumors, 3 showed tumors buds, 5 showed small tumors and only 4 showed big tumors (Figures 1E and 1F). In lung metastasis assays, ASH-WEX fed mice showed strong suppression of metastasis. There were less than 5 tumors in ASH-WEX fed mice as compared to the control mice that had more than 50 tumors (Figure 1G). These data suggested that the ASH-WEX has considerable anticancer activity *in vitro* and *in vivo.*

**Figure 2:**
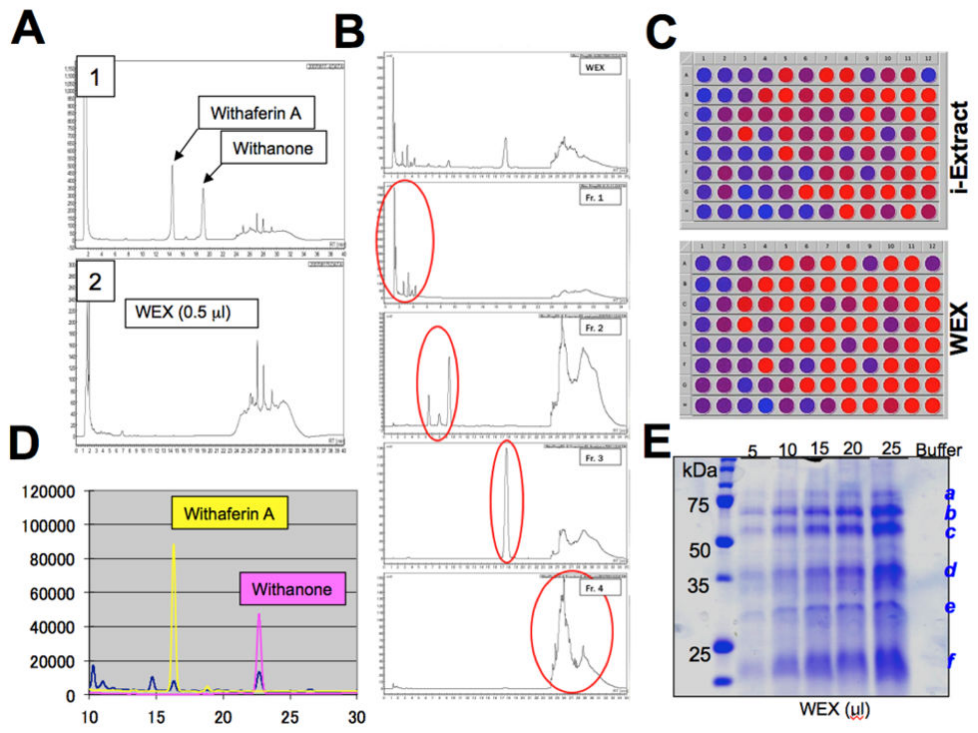
Chemical analysis of WEX (ASH-WEX). (**A**, **B** and **D**) HPLC analysis of WEX under two different conditions showed that WEX has four major components (**B**) and minor amounts of withaferin A and withanone (**D**). Cell-based comparative shRNA-mediated loss-of-function viability analysis of i-Extract and WEX showing that these involve several common gene targets. Blue and Red color in the wells signify dead and live cells, respectively, as examined by Alamar Blue assay (**C**). (**E**) Examination of proteins in the WEX by SDS-PAGE and Coomassie Brilliant Blue staining showing six proteins that acted as a signature of WEX.

 We had earlier identified anticancer activity in the alcoholic extract and its components, withanone. Therefore, we first investigated the presence of withanone in the water extract. HPLC of ASH-WEX was performed at conditions that detected withanone and withaferin A in the alcoholic extract and as described earlier [38]. As shown in Figure 2A-2, there was neither withanone nor withaferin A in the ASH-WEX. Positive control containing the standard solution of these compounds was run in parallel for reference (Figure 2A-1). ASH-WEX, instead, showed the presence of other components that were fractionated under different HPLC gradient conditions as described in the material and methods (HPLC-1). It was seen to have four major fractions as shown in Figure 2B. We compared the cellular targets of ASH-WEX and i-Extract by undertaking cell-based shRNA-mediated loss-of-function screening using 96 shRNA sequences as described earlier [38]. Viability of cells treated with ASH-WEX (1%) and i-Extract (5 µg/ml) was monitored by Alamar Blue Assay. Whereas control cells showed more than 95% cytotoxicity, selected shRNA-transfected cells showed 30-40% viability as indicated by red color in the wells (Figure 2C). These analyses revealed that the i-Extract and ASH-WEX both are cytotoxic to cancer cells and their cytotoxicity could be mediated by some unique and several common gene-targets (Figure 2C), suggesting the similarities in their constituents and mode of action. Based on these findings, we re-examined the HPLC profile of the ASH-WEX under the conditions as described in the material and methods (HPLC-2). Such analysis revealed that the ASH-WEX (100% solution) contained a low level of withaferin A (~20 µg/ml) and withanone (~90 µg/ml) (Figure 2D) that may account for the common gene targets identified in the shRNA screening. Furthermore, SDS-PAGE analysis of the ASH-WEX revealed the presence of 6 proteins labeled as *a-f* (Figure 2E). These features (HPLC profile and proteins) were used as ASH-WEX signature to avoid batch variation during the course of the study. 

**Figure 3:**
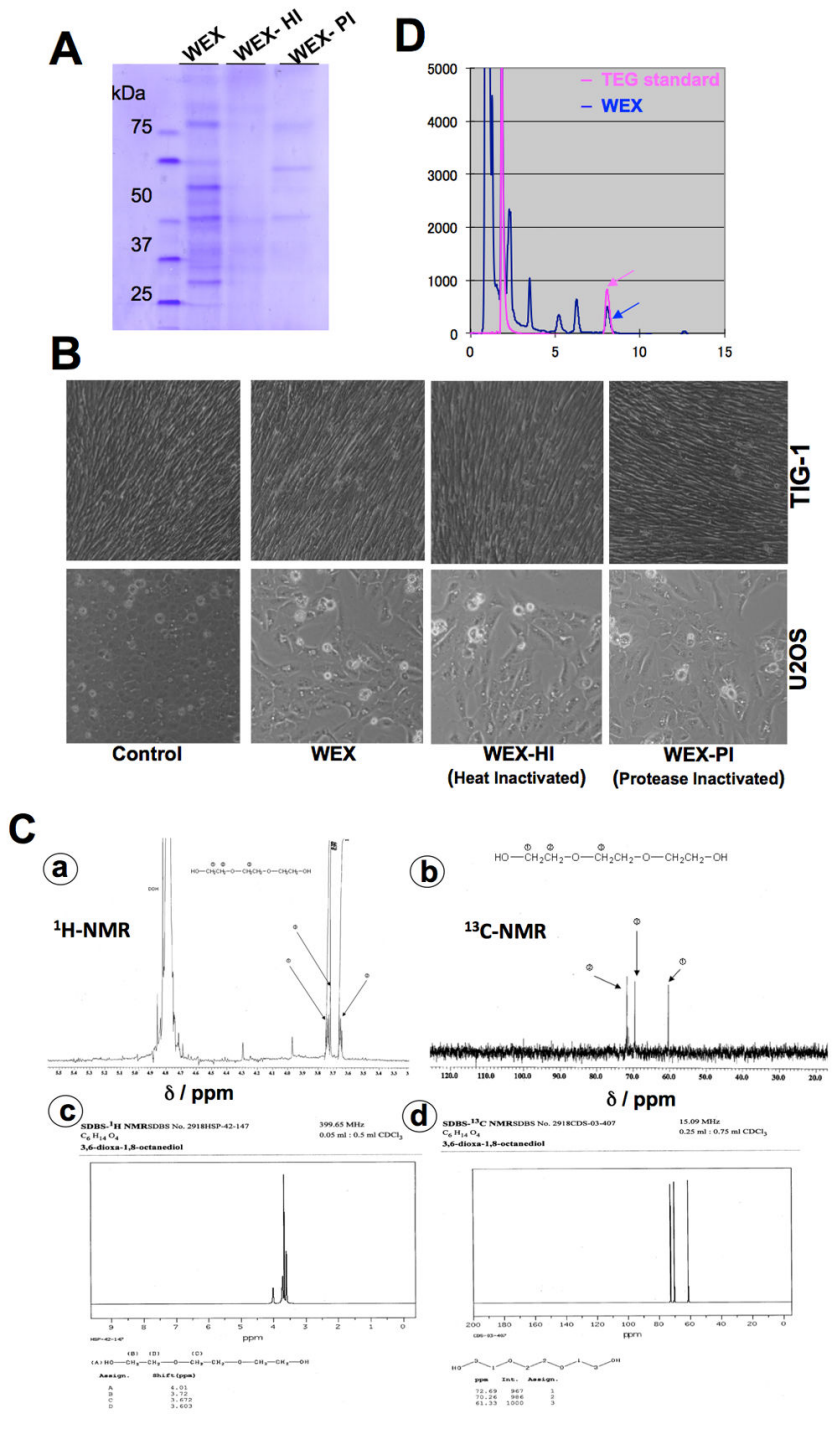
Activity-based chemical identification of anticancer component. (**A**) Heat and proteinase K inactivated WEX (ASH-WEX) as examined on SDS-PAGE. (**B**) Cytotoxicity comparisons of WEX and its heat and protein inactivated fractions showing that the heat and proteinase-K did not alter the activity (**C**) NMR analysis of WEX showing ^1^H and ^13^C spectrums indicating the presence of TEG in WEX (**a** and **b**). NMR spectrums of TEG are shown (**c** and **d**). (**D**) HPLC analysis of WEX to examine the presence of TEG. TEG was used as a standard.

 In order to characterize the anticancer activity in ASH-WEX in cell-based assays, we first inactivated the protein components by heat denaturation and proteinase-degradation (Figure 3A). The assays revealed that the cytotoxic activity of ASH-WEX was independent of its protein components (Figure 3B). Furthermore, it was size fractionated as described in the materials and methods. The active fraction (ASH-WEX-F2, as examined by cytotoxic assays) was heat denatured, dried and subjected to NMR analysis. ^1^H and ^13^C-NMR spectrums (Figure 3C, a-b) predicted the presence of triethylene glycol (TEG) as a major component in ASH-WEX-F2. Indeed, the ^1^H and ^13^C-NMR spectrums of TEG were identical to the ones obtained for ASH-WEX-F2 (Figure 3C, c-d). We performed HPLC of ASH-WEX under conditions as described in the materials and methods (HPLC-3) and using TEG as a standard. These data confirmed the presence of triethylene glycol (TEG) in ASH-WEX (Figure 3D). 

**Figure 4:**
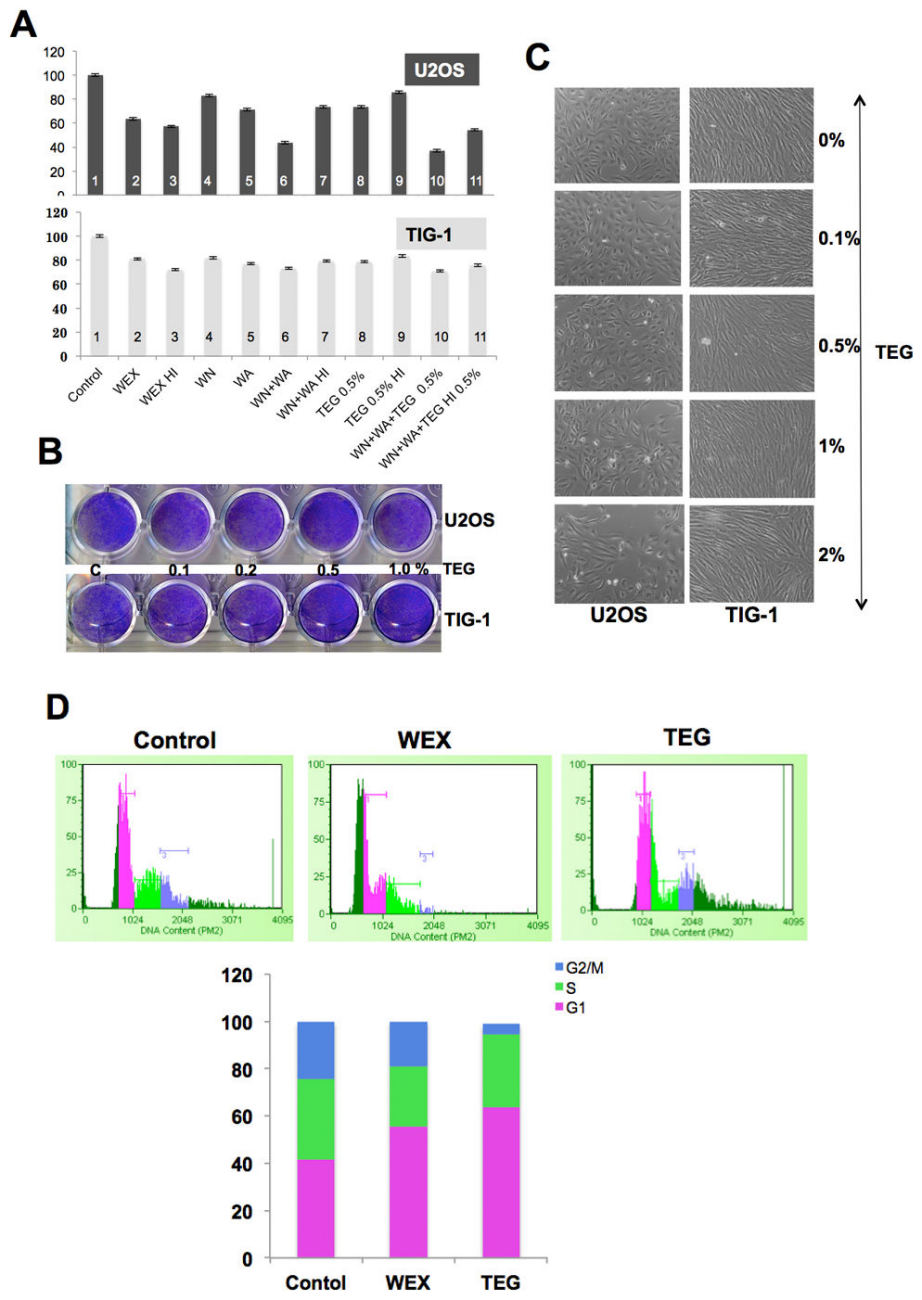
Cancer cell toxicity analysis of TEG. (**A**) Heat inactivated WEX (ASH-WEX), TEG, WA (withaferin A) and WN (withanone) were tested for cancer cell cytotoxicity. WEX and TEG retained their cancer cell toxicity even when inactivated by heat (compare: bars 2 and 3, bars 8 and 9). Statistical significance of the data as calculated by One-way ANOVA test is indicated by * (P<0.01) for control and treated cells, and # (P<0.001) for control and heat inactivated samples. (**B** and **C**) Cancer cells treated with serial doses of TEG for 48-72 h showed decrease in cell number whereas the normal cells showed minor increase. (**D**) Cell cycle analysis of WEX and TEG treated cancer cells revealed increase in number of cells arrested at G1 phase.

 We next investigated whether TEG is the main cytotoxic component of ASH-WEX in *in vitro* and *in vivo* nude mice assays. As described above, in *in vitro* assays, heat inactivation did not cause the loss of cytotoxicity in ASH-WEX. Based on this observation and in order to determine the anticancer component in ASH-WEX, we tested the effect of heat inactivation on cytotoxicity of withaferin A, withanone and TEG. As shown in Figure 4A, cytotoxicity of withaferin A and withanone to U2OS cells was compromised by heat treatment (Figure 4A, compare bar 6 with 7, P value <0.01). However, there was no significant difference in the cytotoxic effect of TEG and heat-inactivated TEG (Figure 4A, compare bar 8 with 9). Furthermore, the cytotoxicity, predominantly seen in cancer cells, was also supported by these assays (Figures 4A and 4B). Normal human fibroblasts treated with serial doses of TEG showed no/minor growth arrest as shown in Figures 4B and 4C. Cell cycle analysis revealed that the ASH-WEX and TEG caused G1 arrest (Figure 4D). 

**Figure 5:**
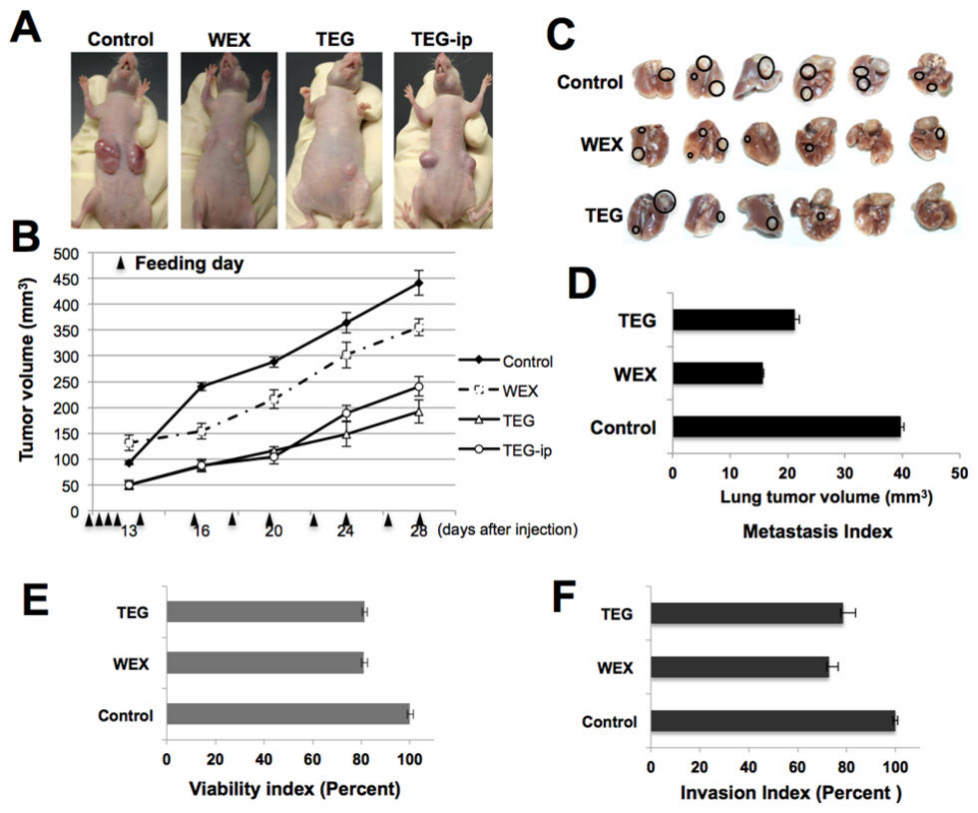
Anticancer activity of TEG in *in* *vivo* assays. (**A**-**B**) Anticancer activity of TEG was examined in nude mice tumor formation assay using HT1080 cells in subcutaneous xenografts and lung metastasis. Both, oral feeding and intraperitoneal injection of TEG, showed suppression in the tumor growth. (**C**) Lung metastasis was significantly reduced in nude mice fed with WEX (ASH-WEX) as well as TEG (**C** and **D**). *In* *vitro* viability and Matrigel Invasion assays on HT1080 cells revealed the reduction in viability as well as invasion capacity (**E** and **F**).

 We next investigated an *in vivo* tumor suppression activity in TEG by examining the nude mice tumor formation and metastasis assays. HT1080 cells that form aggressive tumors with high lung metastasis were used. Tumor volume in control cells showed sharp increase in 28 days. Oral feeding of TEG, similar to ASH-WEX (as described above), to the mice implanted with HT1080 subcutaneous xenograft and tail vein injections showed slow tumor growth as compared to the control mice suggesting tumor suppressive activity in TEG (Figures 5A and 5B). Intraperitoneal injections (100 µl of 5% TEG on every alternate day) also showed similar tumor suppressive effect. Both oral feeding and intraperitoneal injections of TEG were effective to significantly reduce the tumor volume. Furthermore, TEG showed strong anti metastasis activity. All the control mice showed big tumors in the lung. ASH-WEX and TEG-treated mice showed significant reduction in the number and volume of lung tumors (Figures 5C and 5D). The HT1080 cells when treated with either ASH-WEX or TEG showed decrease in viability as well the invasion capacity (Figures 5E and 5F) suggesting that the TEG is a predominant anti-tumor factor in ASH-WEX.

**Figure 6:**
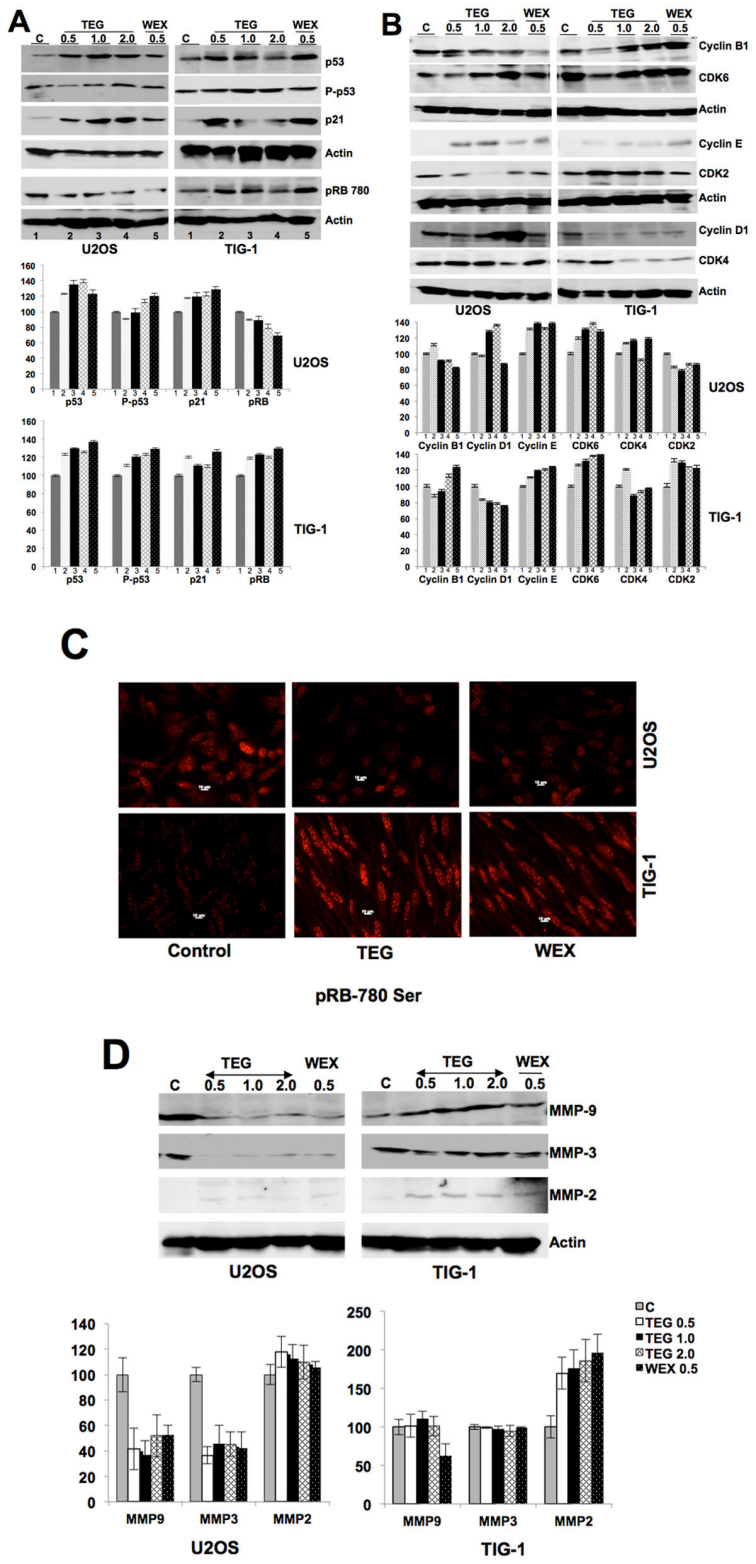
Molecular analyses of the cancer cell specific cytotoxicity and anti-migration activity in WEX (ASH-WEX) and TEG. (**A**) Western analysis of U2OS and TIG-1 cells for indicated proteins. Increase in p53 and p21 was observed both in cancer and normal cells. Whereas phosphorylated RB decreased in cancer cells, it increased in normal cells. Quantitation of the signals from three independent experiments is shown in the lower panel. (**B**) Changes in the level of cyclins (B1, D1 and E), CDK2, CDK4 and CDK6 are shown. Whereas cyclin D1 showed accumulation in cancer cells in response to TEG and WEX treatment; it was decreased in normal cells when treated in parallel. Quantitation of the signals from three independent experiments is shown in the lower panel. (**C**) Phosphorylation of RB as examined by immunostaining is shown. Whereas cancer cells showed decrease in phosphorylated RB, normal cells showed its increase. (**D**) WEX and TEG treated cancer cells, but not the normal cells, showed sharp decrease in MMP-9 and MMP-3. Quantitation of the signals from three independent experiments is shown in the lower panel.

In order to characterize the mechanism of action of TEG cytotoxicity, we treated cancer and normal cells with ASH-WEX and TEG and first examined the expression of major tumor suppressor proteins, p53 and pRB. As shown in Figure 6A, U2OS cells showed increase in p53 when treated with ASH-WEX and TEG. The data was also confirmed by single cell immunostaining of control and treated cells with p53 and its downstream effector, p21 (regulator of growth arrest) (data not shown). Normal cells, however, also showed increase in p53 and p21 when treated with either ASH-WEX or TEG (Figure 6A). Furthermore, phosphorylation of p53, as examined by phosphoserine specific antibody, revealed an increase in phosphorylated p53 protein both in cancer and normal cells. Based on the ratio of phopsho-p53/p53, we found that the ASH-WEX and TEG treated cells showed 30-40% increase in phosphorylation. These data suggested that in contrast to the selective activation of p53 in response to i-Extract treatment as reported earlier [38,42], ASH-WEX caused activation of p53 in cancer as well as normal cells. Investigation on the level of phosphorylated pRB by Western blotting and immunostaining in control (untreated), ASH-WEX and TEG-treated cells revealed that whereas RB phosphorylation decreases in cancer cells, normal cells showed increase in phosphorylated pRB (Figure 6A). Whereas the ratio of phospho RB/RB decreased from ~0.5 in control cells to ~0.3 in TEG-treated cells (20% decrease), similar ratio showed increase in normal cells. In parallel, we also examined the level of cyclin-B1, -D1 and -E1, and CDK-2, -4 and -6. Whereas the treatment of cancer cells with ASH-WEX and TEG showed decrease in cyclin B1, normal cells showed increase in cyclin B1 (Figure 6B). There was a reverse trend in the expression of cyclin D1 when cells were treated with TEG. Whereas cyclin D1 increased in cancer it decreased in normal cells. Cyclin E1 increased in both cancer and normal cells. Moreover, CDK4 was decreased in normal cells and the downstream effector of cyclin-CDK complexes, pRB, showed decrease in phosphorylation in cancer cells (Figure 6C). Normal cells showed increase in phosphorylated RB in parallel treatments (Figure 6C). In order to investigate the mechanism of anti-metastasis activity in ASH-WEX and TEG, we examined the level of expression of matrix metalloproteinases (MMP-3, -9 and -2). As shown in Figure 6D, there was a sharp decrease in the level of expression of MMP-3 and MMP-9 in cancer cells treated with ASH-WEX and TEG, suggesting their anti-metastatic activity as observed in *in vitro* and *in vivo* assays described above. Although there was no change in the expression of MMP-2 in cancer cells, normal cells showed increase in response to ASH-WEX and TEG treatments.

Ashwagandha (root and leaves) has been known as a rich source of withanolides in several studies. These withanolides possess cancer cell cytotoxicity, immunomodulatory and neuroprotective activities [14,17,35,43-47]. To the best of our knowledge, this is the first study reporting the presence of TEG in Ashwagandha leaf extract and its anticancer potential. TEG is a member of dihydroxy alcohol family and is known to have high ability to hold water molecules. It has been well established as a low toxicity mild disinfectant towards a variety of airborne, solution and surface bound microbes including bacteria and viruses [48]. An old study on dogs having inoperable malignant and metastatic tonsillar epithelioma and reticulum cell sarcoma reported the regression of tumors in response to the treatment with TEG without any toxicity [49]. In another study, triethylene tetramine (TETA) was reported as a novel ligand for G-quadruplex that has many kinds of biological activities, including telomerase inhibition and induction of senescence in tumor cells. Furthermore, it was reported that low concentration of TETA had limited ability to inhibit the growth of tumor cells in short-term culture, but it could significantly enhance anti-tumor activity of traditional anti-tumor drugs *in vitro* and *in vivo* [50]. 

We have observed two distinct phenotypes in cancer cells treated with ASH-WEX and TEG. These included (i) growth arrest and (ii) decreased migration and invasion capacity *in vitro* and *in vivo*. Molecular analysis revealed that the tumor suppressor proteins p53 and p21 were induced by approximately 10-20% in response to ASH-WEX and TEG treatments. Furthermore, an increase in phosphorylated p53 suggested that it as a stress response. Examination of normal cells when treated with either ASH-WEX or TEG in parallel revealed similar induction of p53-p21 suggesting that the treatments may evoke a mild stress response both in cancer and normal cells.

 Cyclins are a highly conserved family of proteins that act as regulators of CDK kinases and show dramatic periodicity in their expression level during the cell cycle progression. Different cyclins exhibit distinct expression and degradation patterns that contribute to the temporal coordination of each mitotic event. It has been established that cyclin D1 forms a complex with CDK4 or CDK6 and is absolutely required for G1/S transition. Cyclin D1-CDK4/CDK6 complex forms an active kinase for the retinoblastoma protein (RB) resulting in RB-phosphorylation, release of E2F from RB-E2F complex and transcription of genes required for cell cycle progression. Cyclin D1 also plays a key role in linking the extracellular signaling to cell cycle progression. Its expression level increases during G2 phase, maintains through mitosis and G1 phase, and declines in S phase when DNA synthesis begins. During the cell cycle progression in response to pro proliferative signals, cyclin D1 level gets induced once again during G2 phase and decline on entry to S phase resulting commitment to continuing proliferation. Since ASH-WEX and TEG treatment resulted in G1 arrest in cancer cells, we examined the level of cyclins and CDKs as shown in Figure 6B. When normal and cancer cells were treated with TEG, cyclin D1 showed accumulation only in cancer cells. Furthermore, results from three independent experiments revealed that the level of cyclin B1 decreases in TEG and ASH-WEX treated cancer cells whereas it increases in normal cells. These data were reflective of the predominant growth arrest of cancer cells by ASH-WEX and TEG. To investigate it further, we examined the phosphorylation of RB using phosphor specific antibody. Whereas RB phosphorylation decreased in cancer cells, there was a moderate increase in normal cells (Figures 6B and 6C). Immunocytochemistry with anti-phospho RB antibody confirmed these results (Figure 6C), concluding that the ASH-WEX and TEG cause hypophosphorylation of pRB and activate its tumor suppressor activity. Such selective activation of tumor suppressor RB in cancer cells suggested the differential signaling response of normal and cancer cells to ASH-WEX and TEG. The molecular components and mechanism(s) of this, such as, either differential absorption/intake of the active components or the differential cellular factors/pathways responding to it, warrant further investigations. 

 Antimetastatic activities of TEG and ASH-WEX were supported by expression analysis of matrix metalloproteases (MMP-3, -9 and -2) that revealed remarkable decrease in MMP3 and MMP-9. We have shown, for the first time, that the leaves of Ashwagandha possess TEG that causes selective toxicity to cancer cells by activation of tumor suppressor protein pRB. Since pRB is one of the major tumor suppressor genes, its selective activation in cancer cells may prove highly beneficial for cancer treatment. Ashwagandha leaves thus may provide an easy, economic and safe anticancer remedy.

## Materials and Methods

### Ethics statement

This study was carried out in strict accordance with the recommendations in the Animal Experiment Committee, Safety and Environment Management Division, National Institute of Advanced Industrial Science & Technology (AIST), Japan (Experimental plan approval #2012-025).

### Preparation of water extract of Ashwagandha leaves (ASH-WEX) and its fractions

Ashwagandha plants were raised in Nihari-mura, Ibaraki, Japan by Dr. Takeya Komiya. The leaves were sun-dried and grounded to fine powder. Three kinds of water extracts (10% w/v) were prepared. ASH-WEX-1 was prepared by overnight extraction in sterile water at 40-50°C with slow shaking. For ASH-WEX-2, the leaves-water slurry was microwaved (twice at high 750 W; 3 min each). ASH-WEX-3 was obtained by autoclaving the leaves-water slurry in wet cycle (twice, 20 min each). All the extracts were first set at room temperature and filtered through Whatman filter paper (Grade 4) followed by centrifugation at 10,000 rpm for 20 min and sterile filtration through 0.45 µm filter. ASH-WEX-1 (called ASH-WEX) was used for all the experimentations. ASH-WEX (5% w/v) was also prepared by vigorously stirring the leave powder at room temperature followed by filtration with 2.5 µm filter. The filtrate was subjected to chemical characterization by HPLC. 

### Preparation of ASH-WEX Fractions

For preparation of ASH-WEX fractions, Amicon Ultra-4 Ultracel-10k with 10,000 MWCO (Molecular Weight Cut Off) was used. 3.5 mL of ASH-WEX solution was loaded to the filter device, placed into a fixed rotor centrifuge, and was spun at 7, 500 x *g* for 30 min at 4°C. 0.5 mL of water was added to the device and was centrifuged again for 30 min. The flow through was collected as Fraction 1 (ASH-WEX-F1) (F1-MW<10,000). The residue left on the membrane was collected as Fraction 2 (ASH-WEX-F2) (F2-MW>10,000) by adding small amount of water to the filter device. The fractions was evaporated and weighed. Stock solution (20 mg/ml) was filtered through a 0.45 µm sterile filter, stored at -20°C and used for cell-based assays. ASH-WEX was heated at 99°C for 15 min to make it heat-inactivated (HI). Proteins were inactivated by treating with Proteinase K (20 µg/ml) at 37°C for 30 min. 

### Fractionation and analysis of ASH-WEX by HPLC

 ASH-WEX was fractionated via reversed-phase HPLC using the C18 column (TSKgel ODS-100Z, Tosoh Corporation) (HPLC-1). Reversed-phase HPLC was carried out at a flow rate of 1 ml/min at a column temperature of 40°C. Gradient extraction was carried out with water (Solution A) and ethanol (Solution B). The 35-min gradient program (a constant level of Solution A at 100% for 5 min; gradient to 0.75% of Solution B for 15 min, gradient from 0.75% to 50% of Solution B for 5 min, a constant level of Solution B at 50% for 5 min, gradient from 50% to 0% of Solution B for 2 min, and a constant level of Solution A at 100% for 5 min in the end) was applied. Detection of components in eluted fractions was carried out at 220 nm. ASH-WEX could be fractionated into 4 constituents (Fractions 1 to 4). Symmetry® C-18 (5 μm 150 (l) × 4.6 (d) column was used and fractionation was performed at 40°C using Solution A (1% methanol) and Solution B (methanol: ethanol: isopropanol in ratio of 52.25: 45.30: 2.45) gradient program as follows: Initial - A:B (65:35) followed by 59:41 for 20 min, 0:100 for 23 min, 0:100 for 28 min, 65:35 for 31 min and final elution at 65:35 for 36 min. Flow rate was maintained at 1 ml/min throughout the elution. Detection was performed at 220 nm. 

The second HPLC (HPLC-2) for ASH-WEX and the one prepared at room temperature was performed using Shimadzu HPLC system (LC-2010C). For detection of withanone and withaferin A, ASH-WEX was diluted three times with DMSO and filtered through 0.2 μm filter. Phenomenex HPLC column (Luna 5u C18 (2) 100A :  4.60 mm I.D. x 150 mm) was used and the fractionation was performed at 45°C using Solution A : H_2_O (1% MeOH) and Solution B : methanol: ethanol : isopropanol in ratio of 52.25: 45.30: 2.45) with gradient program as follows.   A: 65 percentage points → 55 percentage points (30 min, flow rate: 1 mL/min; Injection volume: 10 µl). Detection was performed at 220 nm. 

The third HPLC (HPLC-3) for detection of TEG in ASH-WEX was performed with refractive index detector (Shimadzu RID-10A) as reported earlier [51]. Phenomenex HPLC column (Luna 5u C18 (2) 100A:4.60 mm I.D. x 150 mm) was used with H_2_O (injection volume 10 µl, flow rate of 2 mL/min at 40°C) as mobile phase. TEG (Sigma) was used as a standard.

### NMR analysis

ASH-WEX-F2 was dissolved in deuterated water and subjected to ^1^H-NMR and ^13^C-NMR analyses using ECA-500 FT-NMR (nuclear magnetic resonance analysis), JEOL Ltd., Japan. ^1^H-NMR was performed at room temperature at magnetic filed intensity of 11.747T (500 MHz for ^1^H) at the frequency range of 3-15 ppm. 16384 points were collected at the non-decoupling mode of a pulse/7 sec (x 16 times). For ^13^C-NMR, magnetic filed intensity of 11.747T (125 MHz for ^13^C) was used in the frequency range of 25-225 ppm. 32768 points were collected at the decoupling mode of a pulse/2 sec (x 20,000 times)**.**

### Cell culture and treatments

Human osteosarcoma (U2OS) was obtained from American Type Culture Collection (ATCC, Manassas, VA); breast carcinoma (MCF7), fibrosarcoma (HT1080) and normal fibroblasts (TIG, MRC5 and WI-38) were obtained from Japanese Collection of Research Bioresources (JCRB, Japan). Cells were cultured in Dulbecco’s Modified Eagle’s Medium DMEM (Invitrogen)-supplemented with 10% fetal bovine serum in a humidified incubator (37°C and 5% CO_2_). Cells grown at 40-60% confluency were treated with ASH-WEX, its indicated fractions (F1 and F2) and the active component (TEG). ASH-WEX (1% - 200 µg/ml) and its fractions were used for cell treatments in which cells were incubated at 37°C typically for 48 h following which various assays, as described below, were performed.

### Cytotoxicity/growth inhibition assay

Cytotoxicity of ASH-WEX and its different constituents was tested by using WST (2-(2-methoxy-4-nitrophenyl)-3-(4-nitrophenyl)-5-(2,4-disulfophenyl)-2H-tetrazolium), Roche) or MTT {3-(4,5-dimethylthiazol-2-yl)-2, 5-diphenyltetrazolium bromide (Life Technologies) assays in which the cell viability was estimated by the conversion of yellow MTT by mitochondrial dehydrogenases of living cells to purple formazan (MTT assay). MTT (0.5 mg/ml) was added to the cell culture medium for 4 h following the treatment of cells as indicated. MTT containing medium was then replaced with 100 µl of DMSO in each well for complete dissolution of formazan crystals. The absorbance was measured at 550 nm using spectrophotometer (Wallac, ArvoSX).

### Morphologic observations

The cells were cultured at about 50% confluency in 24-well plates. The cells were treated with different concentrations of ASH-WEX and its constituents. After 48 h, morphological changes were recorded by phase contrast microscope (Nikon, Japan). The plates were stained with Crystal Violet and scanned for records.

### Mice toxicity and tumor assays

CD1-ICR mice (6 weeks old, female) and Balb/c nude mice (4 weeks old, female) were bought from Nihon Clea (Japan). Animals used for experimentation received humane care, and all the *in vivo* experiments were performed in accordance with institutional regulations. Mice were housed under pathogen free conditions and under a 12 h dark/light cycle and fed with standard chow *ad libitum*. For toxicity assay, mice were fed with ASH-WEX (200-500 mg ASH-WEX/Kg body weight with silicon needle) every alternate day for 60 days. Body weight of control and ASH-WEX fed mice was recorded every 4 days along with regular observations on their physical activity, eating and drinking habits. For anti-tumor assays, HT1080 cells (6 x 10^6^ cells suspended in 0.2 ml of growth medium) were injected into the nude mice subcutaneously (two sites per mouse) and by tail vein injection (6 x 10^5^ cells suspended in 0.2 ml of growth medium). Control group was treated with 2% carboxymethyl cellulose (CMC), ASH-WEX group was fed with 100-250 mg ASH-WEX/Kg body weight and TEG group was treated with 5% TEG (250 μl for oral feeding and 100 μl for intraperitoneal injections). The treatment started on the 8th day of injection, and was carried out 12 times on alternate days. Tumor formation in the nude mice was monitored every alternate day for a month. Predefined human endpoints were established according to AIST (Japan) Committee on the Ethics of Animal Experiments.  Criteria set for need to euthanize was the tumor size, physical appearance including sickness, distress or immobility. Maximum tumor size allowed was 20 mm at the largest diameter.  None of the animals, in the present study, met any criteria that required euthanization. The volume of subcutaneous tumors was calculated as V=L X W^2^/2, where L was length and W was width of the tumor, respectively. For metastasis assay, the recipient mice were sacrificed by cervical dislocation, lungs were fixed in 4% formaldehyde and the tumor colonies were counted 5 weeks after tail vein injection. This assay was performed using three mice for each group, and repeated twice. 

### SDS-polyacrylamide gel electrophoresis and Western blotting

ASH-WEX (20 μl) was resolved on 10% SDS-PAGE. The gel was stained with Coomassie Brilliant Blue. For biochemical analyses, cells were grown and treated in 6-well plates. After 48 h of treatment, the cells were lysed with RIPA lysis buffer (Thermo Scientific, City). The protein lysate (20 μg, estimated by Bradford method) was separated on SDS-polyacrylamide gel, electroblotted onto a nitrocellulose membrane (Millipore) using a semidry transfer blotter. Immunoassays were done with anti-p53 (DO-1), -p21 (C-19), -CDK6 (C-21), -CDK4 (C-22), -CDK2 (M-2), -MMP9  (C-20), -MMP3 (C-19), -MMP2  (K-20), -cyclin B1 (H-433), -cyclin D1 (A-12), -cyclin E1 (M-20) antibodies (Santa Cruz), anti-γH2AX antibody (Millipore), anti-pRb antibody (ser-780) (Cell Signaling) and anti-actin antibody (Chemicon International, CA). The immunocomplexes formed were visualized with horseradish peroxidase-conjugated anti-rabbit/mouse/goat immunoglobulin G and ECL (Amersham Pharmacia Biotech, NJ). Quantitation of the immunoblots was performed using the ImageJ software (National Institute of Health) and statistical significance was calculated by One-way ANOVA test.

### Immunocytochemistry

Cells were cultured and treated on glass coverslips placed in a 12-well culture dish. At the end of the treatment, coverslips were washed with cold phosphate-buffered saline (PBS) and the cells were fixed with pre chilled methanol:acetone (1:1 v/v) mixture for 5-10 min. Fixed cells were washed with PBS, permeabilized with 0.2% Triton X-100 in PBS for 10 min, and blocked with 2% bovine serum albumin (BSA) in PBS for 20 min. Cells were stained with anti-p53 (DO-1) and -pRb (S780) antibodies. Immunostaining was visualized by secondary staining with Alexa-488 or Alexa-594 conjugated antibodies (Molecular probes). After three to four washings with 0.2% Triton X-100 in PBS (PBST), cells were overlaid with Fluoromount (Difco). The cells were examined under Carl Zeiss microscope with epifluorescence optics. 
