# Correction: Water Extract of Ashwagandha Leaves Has Anticancer Activity: Identification of an Active Component and Its Mechanism of Action

**DOI:** 10.1371/annotation/b7059f27-5970-4734-8601-9913adcce984

**Published:** 2013-11-13

**Authors:** Renu Wadhwa, Rumani Singh, Ran Gao, Navjot Shah, Nashi Widodo, Tomoko Nakamoto, Yoshiyuki Ishida, Keiji Terao, Sunil C. Kaul

An affiliation of the fifth author is incorrect. In addition to institution number 1, Nashi Widodo is not affiliated with institution number 3, but rather institution number 2, Department of Biology, Faculty of Mathematics and Natural

Sciences, Brawijaya University, Malang, Indonesia.

In addition, the affiliation of the seventh and eighth authors is incorrect. Yoshiyuki Ishida and Keiji Terao are not affiliated with institution number 2, but rather institution number 3, CycloChem Co., Ltd. KIBC No. 654 5-5-2 Minamicho Minatoshima Chuo-ku, Kobe City, Japan. 

